# Evaluation of 311 contemporary cases of stereotactic biopsies in patients with neoplastic and non-neoplastic lesions—diagnostic yield and management of non-diagnostic cases

**DOI:** 10.1007/s10143-020-01394-0

**Published:** 2020-09-20

**Authors:** Krystyna Agnieszka Pasternak, Michael Schwake, Nils Warneke, Max Masthoff, Samer Zawy Alsofy, Eric Suero Molina, Walter Stummer, Stephanie Schipmann

**Affiliations:** 1 Department of Neurosurgery, University Hospital Münster, Albert-Schweitzer-Campus 1, 48149 Münster, Germany; 2 Institute of Clinical Radiology, University Hospital Muenster, Münster, Germany; 3 Department of Medicine, Faculty of Health, Witten/Herdecke University, Witten, Germany; 4 Department of Neurosurgery, St. Barbara-Hospital, Academic Hospital of Westphalian Wilhelms-University Münster, Hamm, Germany

**Keywords:** Stereotactic biopsy, Brain lesion, Diagnostic yield, Non-diagnostic biopsy, Glioma, Vasculitis

## Abstract

Stereotactic biopsies are an established tool for obtaining diagnosis of unclear brain lesions. However, non-diagnostic biopsies still occur. We aimed to analyze the contemporary diagnostic yield of stereotactic biopsies, predictors for non-diagnostic biopsies, outcome, and follow-up strategy after non-diagnostic biopsy. We conducted a single-center retrospective study of 311 adult patients undergoing stereotactic biopsies due to a newly diagnosed lesion at our department between 2012 and 2018. Patient data regarding comorbidities, presenting symptoms, imaging features, and non-invasive diagnostic procedures were obtained. The overall diagnostic yield was 86.2% and differed significantly between the various suspected diagnosis groups and was the highest when suspecting primary brain tumor compared with non-neoplastic lesions (91.2% vs. 73.3%, *p* > 0.001). Predicators for non-diagnostic biopsies were small lesion size, lack of contrast-enhancement, presence of sepsis, or underlying hemato-oncological disease. In case of non-diagnostic biopsy, a re-biopsy was performed in 12 cases, revealing a final diagnosis in 75%. In 16 cases, empiric therapy was started based on the suspected underlying disease. Close follow-up was performed in the remaining 15 cases. We showed that stereotactic biopsy is a safe procedure with reasonable diagnostic yield even for non-neoplastic lesions, when non-invasive diagnostic was inconclusive. In addition, we developed treatment recommendations for cases of non-diagnostic biopsies.

## Introduction

Stereotactic biopsy techniques have been widely used since the 1940s and have become an important neurosurgical tool in the diagnosis of intracranial lesions [11]. Apart from primary and secondary brain tumors, lesions can be of inflammatory, infectious, of autoimmune or vascular etiology. Regardless of the etiology, each case demands an accurate and precise histological diagnosis. Many studies demonstrate that stereotactic biopsy techniques enable retrieving tissue even from deep-seated small lesions with comparable low mortality and morbidity [6, 7, 13, 17, 29]. However, with up to 19%, a significant number of non-diagnostic biopsies are reported, especially in patients with non-tumorous lesions [5, 14, 24]. Without obtaining a diagnosis, treatment of the underlying diagnosis is challenging and might be delayed leading to a worse outcome.

We aimed to analyze all cases requiring a stereotactic biopsy at our department regarding diagnostic yield and the underlying etiology of the lesion, paying special attention to factors associated with non-diagnostic biopsies and discuss the outcome and clinical management of those patients.

## Material and methods

We performed a retrospective analysis of all adult patients presenting with newly diagnosed cerebral lesions of unknown etiology that was not amenable to primary surgical resection between January 2012 and December 2018 to our tertiary care neurosurgical department. We enrolled only patients that consecutively were managed by stereotactic needle biopsy.

### Routine management of patients with unclear cerebral lesions

Prior to surgery, all patients presenting with an indeterminate cerebral lesion underwent magnetic resonance (MR) imaging and in selected cases, e.g., when suspecting an underlying low-grade glioma additional 18-F-fluoroethyl-tyrosine (FET)-positron emission tomography (PET) to define hypermetabolic areas.

Each case was discussed by a team of consultant neurosurgeons, neuroradiologists and neurologists, and the attending physician within an interdisciplinary board. In cases of suspected inflammatory disorder or vasculitis, non-surgical tests such as CSF analysis, including microbiologic and virologic analysis, were performed in advance. Only unclear cases or cases where tissue diagnosis was mandatory were scheduled for needle biopsy. A needle biopsy was also performed in patients with tumors that were ineligible for resection due to poor general condition or eloquence of tumor location.

After induction of general anesthesia, a stereotactic head frame was mounted to the head. The stereotactic system was chosen by the attending neurosurgeon according to their preference. The stereotaxic system by KD Lerch (CL Instruments GmbH, Attendorn, Germany) was used between 2012 and 2014 and replaced by Leksell (Elekta, Stockholm, Sweden) and ZD (Zamorano-Duchovny) stereotactic system (Inomed GmbH, Emmendingen, Germany) after 2014.

A 1-mm slice contrast-enhanced CT scan was subsequently acquired. Needle trajectory, entry point, and biopsy targets were determined using iPlan Stereotactic planning software Version 3.0 (BrainLab AG, Munich, Germany). Preoperative MRI (0.6 mm MPR T1Gd or FLARI) and PET images, as available, were fused with the acquired CT scan for precise target and safe entry point selection. A contrast-enhanced region of the lesion—if applicable—was targeted. The stereotactic system was mounted and a 3-cm scalp incision followed by a single burr hole was placed at the planned entry site. Depending on the size of the lesion, approximately 10-mm-long and 1.5-mm-thick serial tissue biopsies with 2.1-mm needles (Neuromedex, Hamburg, Germany) were collected throughout the lesion. In case of suspected vasculitis, additional biopsies were taken from dura, arachnoid, and cortex. We aimed to use a trajectory that is as vertical as possible to cortex and lesion and hereby sparing sulci, blood vessels, and ventricles.

For frameless stereotactic biopsy, a 3-point Mayfield clamp was fastened to the head and the BrainLab VarioGuide System and software (iPlan 3.0 and Elements, BrainLab AG, Munich, Germany) were used as previously described [10]. For deep-seated lesions, frames were used, whereas for more superficial lesions, the attending surgeon could choose between a frame-based or frameless procedure.

After the procedure, patients were observed for neurological deterioration in the intensive care unit. A postoperative cranial CT scan was performed the following day to confirm location of the biopsy and to rule out hemorrhage.

### Patient data

The electronic medical records of each patient were reviewed and baseline demographic data as well as patients’ comorbidities, presenting symptoms, surgical characteristics, and laboratory features were obtained. In addition, lesion features such as depth, size, and location, e.g., multifocality and radiological features such as contrast enhancement and presence of edema were assessed during reviews of imaging by two independent authors.

Cerebrospinal fluid (CSF) was obtained in cases with suspected inflammatory disorder or in case of lymphoma prior to biopsy. Abnormal results were defined as elevated cell count, protein elevation, presence of pathogens, or pathological cells. Neuropathological and, if appropriate, microbiological diagnoses were obtained from biopsies. Neuropathological diagnosis was reported according to the criteria of the 2016 World Health Organization (WHO) classification of Central Nerve System (CNS) tumors [20].

The primary outcomes were diagnostic yield, representing the percentage of cases with a definitive neuropathological diagnosis after biopsy, and procedure-related morbidity and mortality. In addition, in case of non-diagnostic biopsies, the subsequent clinical course and treatment strategies were evaluated.

Informed consent was obtained from each patient. All procedures performed in studies involving human participants were in accordance with the ethical standards of the institutional research committee (Ref 2019-379-f-S) and with the 1964 Helsinki declaration and its later amendments or comparable ethical standards.

### Statistical analysis

For statistical analysis, IBM SPSS Statistics 25.0 software (IBM, Armonk, NY, USA) was used. Data was described by standard statistics, using absolute and relative frequencies for categorical variables and median with interquartile range for continuous variables.

Chi-square test and *t* test or Mann-Whitney *U* test were used for categorical and continuous variables, respectively. All factors that showed statistical significance in univariate analysis were combined in a multivariate logistic regression model. Odds ratios (OR) were obtained with corresponding 95% confidence intervals (CI). Patients with missing information about one variable were *only* excluded from the *corresponding* statistical analyses but not from the entire study. Statistical significance was defined as the probability of a type one error below 5% (*p* < 0.05).

## Results

### Patient data

A total of 311 patients diagnosed with an unclear cerebral lesion underwent stereotactic surgery and were included into analysis. Mean patient age was 61 years (IQR 24), 55.6% (173/311) were male, and 44.4% (138/311) were female. Most patients presented with motor deficits (35.4%, 110/311) or cognitive deficits (27.3%, 85/311); in the majority of cases (43.5%, 131/301), symptom onset was more than 1 month prior to presenting to our department. Lesions were deep seated (thalamus, brain stem, midline structure, basal ganglia) in 17% (53/311).

In almost half of all cases, the suspected diagnosis was primary brain tumor (47.6%, 148/311). Prior to needle biopsy, one-quarter of all patients (76/311) received steroids. Further baseline characteristics regarding comorbidities and imaging features of the suspect cerebral lesions are listed in Table 1.

**Table 1:** Baseline characteristics of all cases and stratified into diagnostic and non-diagnostic biopsy results

		All cases *n* = 311 (%)	Diagnostic-biopsy *n* (%)	Non-diagnostic biopsy *n* (%)	*p* value
Age	*Median, IQR*	61 (24)			0.005
	18–60 years	148 (47.6)	118 (79.7)	30 (20.3)	0.002
	> 60 years	163 (52.4)	150 (92.0)	13 (8.0)	
Sex	Male	173 (55.6)	150 (86.7)	23 (13.3)	0.761
	Female	138 (44.4)	118 (85.5)	20 (14.5)	
Suspected diagnosis	Primary brain tumor	148 (47.6)	135 (91.2)	13 (8.8)	< 0.001
	Lymphoma	73 (23.5)	63 (86.3)	10 (13.7)	
	Other tumor	12 (3.9)	11 (91.7)	1 (8.3)	
	Inflammatory (autoimmune)	15 (4.8)	12 (80.0)	3 (20.0)	
	Inflammatory (infectious)	28 (9.0)	24 (85.7)	4 (14.3)	
	Vascular	17 (5.5)	8 (47.1)	9 (52.9)	
	Unclear	18 (5.8)	15 (83.3)	3 (16.7)	
Comorbidities	Diabetes mellitus	43 (13.8)	38 (88.4)	5 (11.6)	0.653
	Nicotine abuse	31 (10.0)	24 (77.4)	7 (22.6)	0.137
	Alcohol abuse	7 (2.3)	7 (100)	0 (0.0)	0.284
	Drug abuse	2 (0.6)	1 (50.0)	1 (50.0)	0.137
	HIV	2 (0.7)	0 (0.0)	2 (100)	< 0.001
	Hepatitis B	2 (0.7)	2 (100)	0 (0.0)	0.571
	Hepatitis C	1 (0.4)	1 (100)	0 (0.0)	0.694
	Epilepsy	25 (8.0)	20 (80.0)	5 (20.0)	0.351
	Solid malignant tumor	34 (10.9)	30 (88.2)	4 (11.8)	0.712
	Hemato-oncological disease	30 (9.6)	22 (73.3)	8 (26.7)	0.032
	Chemotherapy (within last 3 months)	8 (2.6)	6 (75.0)	2 (25.0)	0.354
	Radiotherapy (within last 3 months)	1 (0.3)	0 (0.0)	1 (100)	0.012
	Autoimmune disease	17 (5.5)	13 (76.5)	4 (23.5)	0.233
	Immunosuppression	11 (3.5)	6 (54.5)	5 (45.5)	0.002
	Sepsis	12 (3.9)	8 (66.7)	4 (33.3)	0.046
Presenting symptoms	Headache	36 (11.6)	31 (86.1)	5 (13.9)	0.991
	Dizziness	33 (10.6)	26 (78.8)	7 (21.2)	0.194
	Impaired vigilance	18 (5.8)	14 (77.8)	4 (22.2)	0.288
	Seizure	53 (17.0)	52 (98.1)	1 (1.9)	0.006
	Cranial nerve dysfunction	35 (11.3)	31 (88.6)	4 (11.4)	0.663
	Motor deficits	110 (35.4)	93 (84.5)	17 (15.5)	0.538
	Sensory deficits	27 (8.7)	21 (77.8)	6 (22.2)	0.186
	Cognitive deficits	85 (27.3)	74 (87.1)	11 (12.9)	0.782
	Aphasia	54 (17.4)	44 (81.5)	10 (18.5)	0.272
	Elevated intracranial pressure	13 (4.2)	11 (84.6)	2 (15.4)	0.868
	Incidental finding	6 (1.9)	6 (1000)	0 (0.0)	0.322
Symptom onset	Peracute (1–2 days)	27 (9.0)	24 (88.9)	3 (11.1)	0.970
	Acute (3–10 days)	55 (18.3)	47 (85.5)	8 (14.5)	
	Subacute (11–30 days)	88 (29.2)	75 (85.2)	13 (14.8)	
	Chronic (> 1 month)	131 (43.5)	70 (85.5)	11 (14.5)	
Treatment before biopsy	Steroids	76 (24.4)	63 (82.9)	13 (17.1)	0.194
	Immunosuppressants	6 (1.9)	1 (16.7)	5 (83.3)	< 0.001
	Anti-infective therapy	33 (10.6)	26 (78.8)	7 (21.2)	0.301
Side of pathology	Right	95 (30.5)	83 (87.4)	12 (12.6)	0.533
	Left	95 (30.5)	84 (88.4)	11 (11.6)	
	Bilateral	121 (38.9)	101 (83.5)	20 (16.5)	
Distribution of the lesion	Supratentorial	293 (94.2)	252 (86.0)	41 (14.0)	0.892
	Infratentorial	1 (0.3)	1 (100)	0 (0.0)	
	Supratentorial and infratentorial	17 (5.5)	15 (88.2)	2 (11.8)	
Region	Frontal	52 (16.7)	44 (84.6)	8 (15.4)	0.245
	Parietal	57 (18.3)	50 (87.7	7 (12.3)	
	Temporal	48 15.4)	38 (79.2)	10 (20.8)	
	Occipital	25 (8.0)	24 (96.0)	1 (4.9)	
	Brainstem, diencephalon, mesencephalon, midline structures	24 (7.7)	22 (91.7)	2 (8.3)	
	Basal ganglia	14 (4.5)	13 (92.9)	1 (7.1)	
	Thalamus	15 (4.8)	15 (100)	0 (0.0)	
	Multiple regions	76 (24.4)	62 (81.6)	14 (18.4)	
Size	< 1 cm^3^	22 (7.1)	9 (40.9)	13 (59.1)	< 0.001
	> 1 cm^3^	289 (92.9)	259 (89.6)	30 (10.4)	
Unilocular/multilocular	Unifocal	211 (67.8)	187 (88.6)	24 (11.4)	0.069
	Multifocal	100 (32.2)	81 (81.0)	19 (19.0)	
Contrast enhancement	Present	247 (79.4)	223 (90.3)	24 (9.7)	< 0.001
Perilesional edema	Present	236 (75.9)	217 (91.9)	19 (8.1)	< 0.001
Laboratory results before biopsy	Abnormal CSF findings (lab)	75 (65.8)	53 (69.3)	23 (30.7)	0.143
	Abnormal CSF (neuropathology)	23 (40.4)	21 (91.3)	2 (8.7)	0.149
	Abnormal CSF (microbiology)	2 (5.1%)	2 (100)	0 (0)	0.394
	Abnormal CSF (virology)	10 (12.8)	6 (60.0)	4 (40.0)	0.265
Stereotactic system	KD Lerch	90 (28.9)	71 (78.9)	19 (21.1)	0.023
	Leksell	112 (36.0)	103 (92.0)	9 (8.0)	
	VarioGuide	76 (24.4)	63 (82.9)	13 (17.1)	
	ZD	33 (10.6)	31 (93.9%)	2 (6.1)	

*IQR*, interquartile range; *CSF*, cerebrospinal fluid; *HIV*, human immunodeficiency virus; *ZD*, Zamorano-Duchovny

### Pathological diagnoses and diagnostic yield

An overview of all neuropathological diagnoses of biopsies is given in Table 2. The main diagnosis was primary brain tumor in almost half of all cases (46.6%, 145/311). Grade IV, III, and II gliomas were reported in 69/145, 36/145, and 36/145 primary brain tumor cases, respectively. Considering the relevant molecular determinants for glioma from the new WHO brain tumor classification, IDH status was characterized in 83.4% (121/145) of all glioma cases and MGMT in 82.6% (90/109) of high-grade gliomas. When considering only biopsies performed after 2015, after introduction of the revised WHO classification [20], IDH was obtained in 96.9% (94/97) and MGMT in 92.8% (64/69) of high-grade glioma cases. The remaining cases did not allow MGMT and IDH analysis.

**Table 2:** Histopathological diagnosis of the 311 brain biopsies

Neuropathological diagnosis		*n* (%)
Primary glial brain tumor		145 (46.6)
	Glioblastoma WHO IV	69
	Anaplastic astrocytoma WHO III	36
	Diffuse astrocytoma WHO II	36
	Diffuse midline glioma WHO IV	4
Molecular diagnosis in glioma patients	IDH status obtained	121 (83.4)
	Wildtype	100
	Mutated	21
	MGMT status obtained*	90 (82.6)
	MGMT unmethylated*	41
	MGMT methylated*	49
	1p/19q codeletion status obtained	11 (7.6)
	1p/19q codeletion	0
Lymphoma		51 (16.4)
	Primary CNS lymphoma (DLBCL)	45
	Secondary CNS lymphoma	6
Other tumors		9 (2.9)
	Metastasis	4
	Histiocytosis	2
	Germial tumor	2
	Meningioma	1
Vascular		18 (5.8)
	Vasculitis	6
	Old hemorrhage/infarction	10
	Amyloid angiopathy	2
Inflammatory (infectious)		30 (9.6)
	Abscess	19
	PML	4
	Opportunistic infection	1
	Encephalitis	6
Inflammatory (autoimmune)		15 (4.8)
	CNS degenerative diseases	8
	Encephalitis	7
Non-diagnostic	Unspecific reactive changes	43 (13.8)

*PML*, progressive multifocal leukoencephalopathy; *CNS*, central nervous system; *MGMT*, O6-methylguanine–DNA methyltransferase; *DLBCL*, diffuse large B cell lymphoma; *IDH*, isocitrate dehydrogenase

“*” refers only to high-grade gliomas (*n* = 109)

**Fig. 1:**
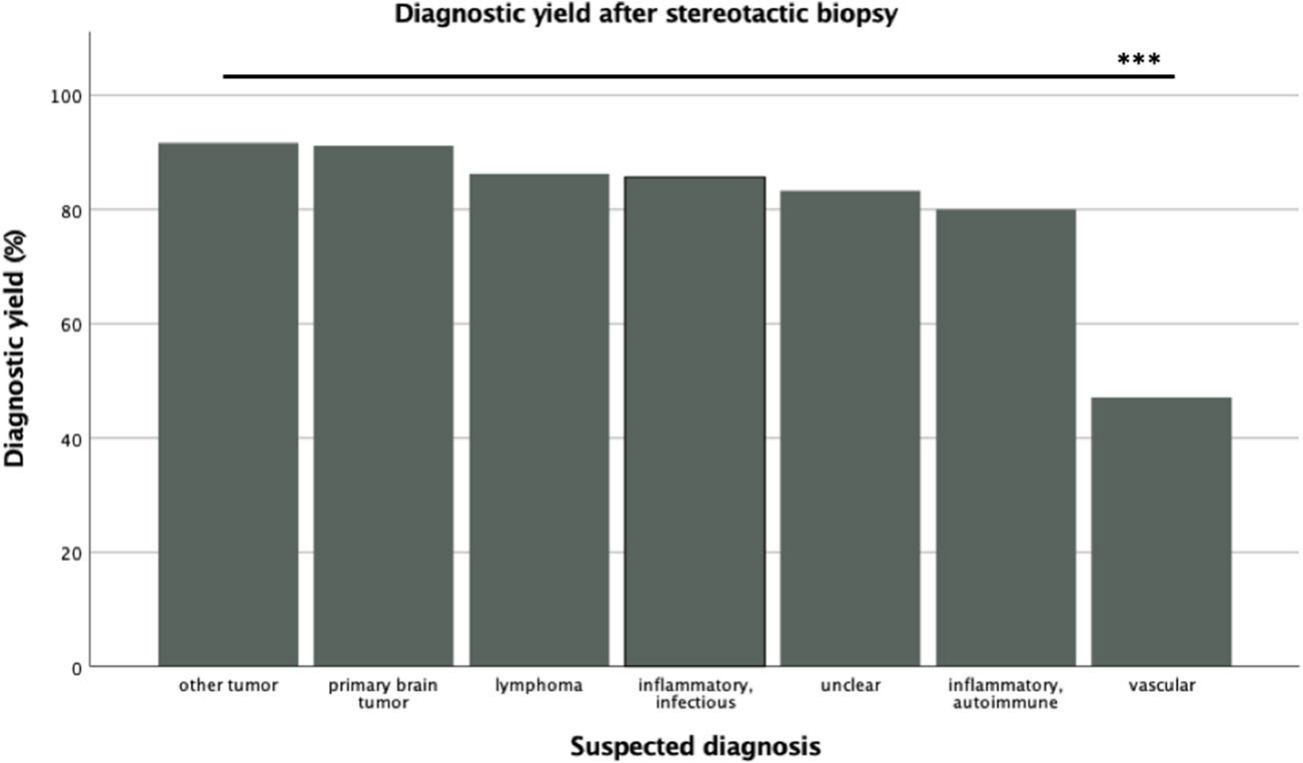
Diagnostic yield with regard to the different underlying suspected diagnoses. ***p < 0.001

The samples were sufficient for diagnosis in 268 cases, giving an overall diagnostic yield of 86.2% (268/311). The diagnostic yield differed significantly between the various diagnosis subgroups and was highest in case of suspected primary and other brain tumors (91.2%, 135/148, and 91.7%, 11/12, respectively) and lowest in case of suspected underlying vascular disease, e.g., vasculitis (47.1%, 8/17) (*p* < 0.001, Fig. 1). The diagnostic yield was slightly higher with frame-based (87.2%, 205/235) than in the frameless (82.9%, 63/76) procedures, without reaching statistical significance (*p* = 0.341). When comparing all stereotactic systems used over time, biopsies obtained with the ZD or Leksell stereotactic systems were significantly more likely to be diagnostic (93.9%, 31/33 vs. 92.0%, 103/112, respectively) than using VarioGuide or KD Lerch system (82.9%, 63/76 vs. 78.9%, 71/90, respectively) (*p* = 0.023) (Table 1). The initially suspected diagnosis was confirmed in 203/311 cases (65.3%).

### Factors associated with a non-diagnostic biopsy

In patients that suffered an underlying hemato-oncological disease and that were treated for sepsis, a non-diagnostic biopsy was more likely (Table 3). In contrast, larger lesions (> 1cm^3^) were 7.5-fold and contrast-enhancing lesions almost 5-fold more likely to be diagnostic. The highest diagnostic yield could be achieved using the ZD stereotactic system (OR: 8.97 95% CI: 1.45–55.63, *p* = 0.018).

**Table 3:** Multivariate logistic regression model predicting a diagnostic sample

Diagnostic biopsy Included variables: age, hemato-oncological disease, sepsis, immunosuppression, contrast-enhancing lesion, perilesional edema, size of the lesion, suspected diagnosis, stereotactic system (KD Lerch, Leksell, VarioGuide, ZD)					
		OR	95% CI		*p* value
Hemato-oncological disease	Yes	Ref			
	No	4.42	1.01	19.35	0.049
Sepsis	Yes	Ref			
	No	19.68	2.55	152.11	0.004
Contrast-enhancing lesion	Yes	4.93	1.54	15.76	0.007
	No	Ref			
Perilesional edema	Yes	3.23	1.07	9.71	0.037
	No	Ref			
Size of lesion	≤ 1 cm^3^	Ref			
	> 1 cm^3^	7.51	2.07	27.18	0.002
Stereotactic system	KD Lerch	Ref			
	Leksell	5.89	1.94	17.79	0.002
	VarioGuide	2.34	0.82	6.68	n.s.
	ZD	8.97	1.45	55.63	0.018

*OR*, odds ration; *95% CI*, 95% confidence interval; *Ref*, reference; *ZD*, Zamorano-Duchovny

### Complications and 30-day mortality

Postoperative complications occurred in 21 of 311 cases (6.8%); the main complication was the onset of a new neurological deficits (5.5%, 17/311), in some cases attributed to symptomatic intracranial hemorrhage (2.9%, 9/211), requiring re-operation. The 30-day mortality rate was 4.5% (14/311); however, only two of them (0.6%) were procedure-related (Table 4). The rate of a persisting neurologic deficit > 30 days was 3.9% (12/311). In a total of 248 cases, a routine postoperative CT was performed, showing, non-symptomatic hemorrhage in 26/248 (10.5%) of cases.

**Table 4:** Complications and mortality rate after stereotactic biopsy

Type of complication		*n* (%)
Complication		*21 (6.8)*
	Symptomatic hemorrhage	9 (2.9)
	Surgical site infection	1 (0.3)
	New neurological deficit	17 (5.5)
	Decompensation of underlying pathology, massive cerebral edema	5 (1.6)
	Reoperation due to complication	9 (2.9)
30-day mortality		*14 (4.5)*
	Procedure-related mortality	2 (0.6)

### Non-diagnostic lesions

**Fig. 2:**
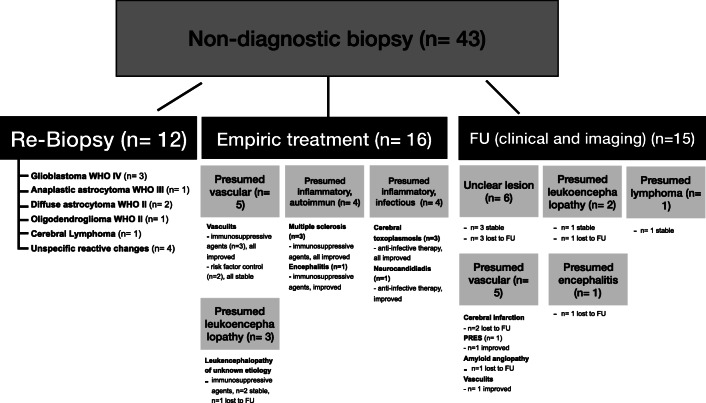
Overview on management after non-diagnostic biopsy

We identified 43/311 (13.8%) cases of non-diagnostic biopsies showing only reactive brain tissue in histopathological analysis despite immunohistochemical evaluations for IDH, MIB (Ki-67), or p53. Follow-up and outcome of these cases are presented in Fig. 2 and cases can be divided into three groups. Concordance between the definitive and intended biopsy localization was documented in 34/43 (79.1%) cases on postoperative CT scans. The remaining cases did not receive a postoperative scan.

#### Re-biopsy

A re-biopsy was performed in 12/43 cases (27.9%), revealing a definitive diagnosis of primary brain tumor (7/12) and lymphoma (1/12) in 75%. These 8/12 cases with initial non-diagnostic biopsy and diagnostic result in repeat biopsy were subject to further analyses regarding reasons for non-diagnostic biopsy. In 5/7 cases (71.4 %) that were diagnosed as glioma in repeat histology, the tumor infiltrating zone was presumably biopsied as initial histology revealed slightly higher proliferation rate and chronic inflammation without clear evidence for tumor cells. This could also be confirmed by postoperative MRI imaging, visualizing the biopsy localization. In two of these 7 cases (2/7, 28.6%), an open biopsy was performed, whereas in 5/7 cases (71.4%), the stereotactic biopsy was repeated. In the remaining glioma case (1/7), the target was initially missed with frameless technique and biopsy was repeated using a frame-based stereotactic procedure.

The first biopsy of the patients with later diagnosed lymphoma revealed reactive brain tissue while the postbiopsy CT indicated a correct localization of the biopsy. The second biopsy was taken from the other side where a clear tumor progress was seen after a few weeks, while the initial biopsy place remained without evidence for tumor progression. Diagnosis of lymphoma was delayed as the re-biopsy was performed 184 days after initial non-diagnostic biopsy, whereas the re-biopsy in case of primary brain tumor was performed within 4 weeks after initial biopsy (median 16 days, range: 9–27).

Taken together, the reasons for these unclear biopsies can be classified as a target error.

In the remaining 4/12 cases (25%), a repeat biopsy could not confirm a diagnosis despite verification of correct biopsy localization on MRI in 2 cases and CT in 2 cases; both biopsies resulted in reactive brain tissue. No progression of the underlying lesion in these cases was observed during further follow-up.

There were no differences regarding application of steroids related to the validity of the biopsy in all mentioned cases.

#### Empiric treatment

Based on the presumed underlying diagnosis (vascular, inflammatory, leukoencephalopathy), derived from clinical and additional laboratory and imagining findings, empiric treatment, using immunosuppressive agents and medication to control risk factors—as appropriate—and additional serial imaging were initiated in 16/43 cases (37.2%). None of these patients worsened clinically during a median follow-up of 37 months (range: 5–80) and symptoms improved in 11/16 cases (68.8%). One case was lost to follow-up.

#### Follow-up (FU)

In the remaining 15/43 cases (34.9%), clinical follow-up and serial imaging were performed over a median follow-up time of 24 months (range: 1–88). 8/15 cases were lost to follow-up. In no case, a progression of the underlying disease occurred during follow-up. No specific treatment was necessary to maintain the good neurological status of these patients.

## Discussion

We performed an analysis of the diagnostic yield, outcome, and management in 311 patients after stereotactic biopsy for an intracranial lesion with special regard to predictors for non-diagnostic biopsies.

### Diagnostic yield according to suspected diagnoses

The overall diagnostic yield in our study was 86.2%, which, at first glance, appears to be low in comparison with other recently published series, ranging between 88.1 and 98.2% [1, 4, 12, 17, 23]. However, we included a significant number of patients with non-neoplastic lesions. Our data revealed that the frequency of non-diagnostic biopsies was higher among patients with non-neoplastic lesions (89.7% for neoplastic lesions vs. 73.3% for non-neoplastic lesions, *p* = 0.001). These findings are in accordance with the current literature [5, 15, 21, 28]. Often brain biopsies are requested by neurologists as an ancillary procedure, e.g., in cases of unclear neurological decline and inability of non-invasive investigations to yield in a diagnosis or failure of empiric treatment strategies. In our series, 17 patients underwent stereotactic brain biopsy, suspecting vasculitis. We found a diagnostic yield of 47.1% in comparison with other studies that report frequencies of 36% [3].

Although the diagnostic yield for non-neoplastic lesions is comparably low, it still reaches 73.3% in our series. Considering the low morbidity and mortality observed in our study and demonstrated by various other publications [6, 7, 16, 27, 29], the propensity of this procedure for establishing a diagnosis in a high percentage of such cases with unclear neurological deterioration justifies performing a brain biopsy, after exploiting all non-surgical diagnostic tests. This concept is supported by other groups [24, 30].

### Predictive factors for diagnostic yield and non-diagnostic biopsies

In order to further improve the diagnostic yield, we aimed at analyzing predictors for non-diagnostic biopsies and found several factors that influence the likelihood of a non-diagnostic probe.

The presence of an underlying hemato-oncological disorder, e.g., leukemia (OR: 4.4) and sepsis (OR: 19.7) was strongly associated with non-diagnostic biopsies. This patient group is more likely to have non-neoplastic cerebral lesions associated with unspecific inflammatory reactions, which is challenging to be detected via small core biopsies [21]. This stands in contrary to the findings of a study on pediatric patients with cryptogenic brain lesions and a study analyzing patients with neurological diseases of unknown etiology, revealing that immunocompromised patients were more likely to yield a diagnosis at biopsy [18, 22, 26]. Radiological features such as the presence of contrast enhancement (OR: 4.9) and perilesional edema (OR: 3.2), as can be observed in most malignant neoplastic lesions or abscesses, were found to be associated with a higher likelihood of a diagnostic biopsy. Lara-Almunia et al. confirmed the presence of contrast-enhancement as a predictor for a diagnostic biopsy. However, they report the presence of significant edema as being associated with a non-diagnostic probe [17] which we could not confirm in our cohort.

In addition, our analysis showed that it is more likely to obtain a diagnostic biopsy from a lesion with a size of more than 1 cm^3^ than from smaller lesions (OR: 7.5). These data are confirmed by other studies [21, 28, 29, 31, 32]. Maragkos et al. showed in their cohort of 198 patients that for every additional mm of lesion diameter, the odds of yielding a diagnostic sample increases by 94% [21]. Smaller lesions are more likely to be missed by the surgeon and handling and analysis of limited tissue probes are challenging for the neuropathologists [21, 29]. It has been shown that overall diagnostic accuracy achieved on histopathology correlates with the amount of tissue obtained during biopsy [14].

We found mainly non-modifiable variables to be associated with a non-diagnostic probe. However, other modifiable predictors, such as performance of intraoperative frozen section examinations [15], MRI- [9], and FDG-PET-guided biopsies [17, 19] are reported in the literature. Some studies reported the neurosurgeon`s experience as a pre-eminent predictive factor for diagnostic yield [17, 25] and revealed an impact of the anatomic location [7, 31].

The only modifiable factor in our study was the choice of the frame. We reached the lowest rate of non-diagnostic biopsies using ZD stereotactic system, which might also be biased by other factors as the choice and experience of the individual surgeon. However, we observed no significant difference regarding the use of frameless or frame-based systems, which is in accordance with the literature [7, 14, 31, 32] and a recently published meta-analysis [8].

We routinely take biopsies from multiple points along the biopsy trajectory, always balancing a potential increase in diagnostic yield against a possible increased risk of neurological deficit. However, our data did not allow analyzing the impact of the number of biopsies on the diagnostic yield. We believe that obtaining tissue from more than one target might improve diagnosis especially in heterogenous lesions.

### Complications and 30-day mortality

We noted a procedure-related mortality rate of 0.6% and morbidity rate of 6.8%. These results are comparable with those reported in the literature, where the morbidity rate ranges between 0.5 and 13% and the mortality rate between 0 and 4%, respectively [6, 7, 16, 26, 27, 29].

Still, there is a non-deniable rate of morbidity after stereotactic biopsy that has to be balanced against the potential gain regarding a diagnosis especially in non-neoplastic cases or cases that have a higher risk for non-diagnostic biopsy. Biopsies in such cases should only be performed after extensive conventional and non-invasive diagnostic procedures have proved to be inconclusive.

### Management of patients with non-diagnostic biopsies

Despite paying attention to factors that are associated with non-diagnostic biopsies during surgical planning, there are still cases that are left without revealing a diagnosis.

**Fig. 3:**
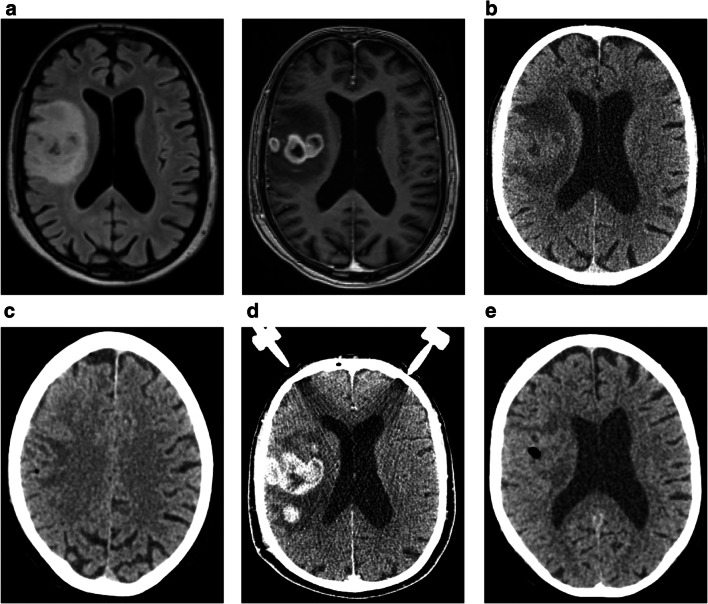
Patient was admitted to neurosurgery with FLAIR hyperintense lesion (**a**, left panel) with partial ring enhancement and perifocal edema (**a**, right panel) highly suggestive for high-grade brain tumor. Biopsy planning included CT-based navigation (**b**). CT after first biopsy reveals small air bubble representing sample location mostly within perifocal edema (**c**). Since first biopsy result was non-conclusive, re-biopsy was planned using contrast-enhanced CT with stereotactic frame (**d**). CT after second biopsy shows a more central sampling as suggested by an according air bubble (**e**). Second biopsy revealed glioblastoma as diagnosis

There is still a lack of treatment and management paradigm for such cases [2, 32]. In total, 43 patients (13.8%) that differed regarding their suspected underlying disease and clinical presentations revealed a non-diagnostic biopsy result. Regarding further management, these patients can be divided into three groups. In 12 cases (27.9%), a re-biopsy was performed due to highly suspected tumor (Fig. 3). In 8 of these 12 cases (66.7%), a diagnosis could be obtained and initial biopsy underlay a target error. In 4 cases (33.3%), the repeat biopsy remained non-diagnostic. However, a close follow-up of these patients did not show any signs of clinical or morphological progression of the underlying lesion.

**Fig. 4:**
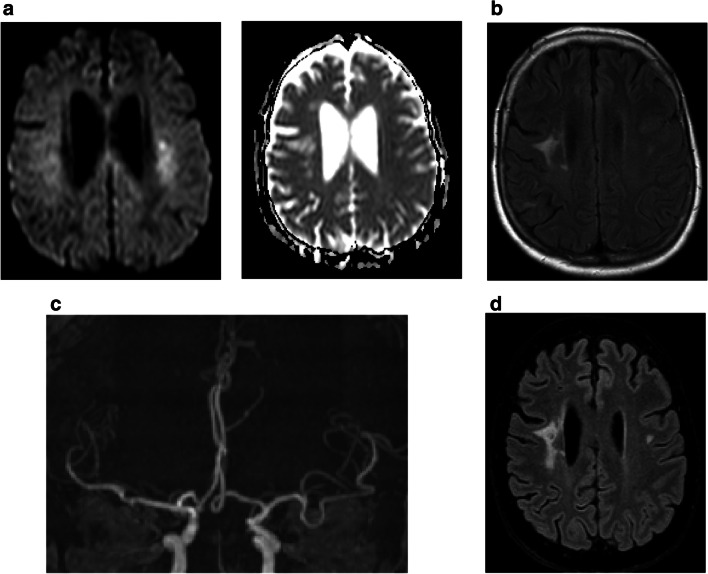
Patient was admitted with suspected cerebral vasculitis. MRI showed small restriction in diffusion within the left corona radiata (**a**, DWI left and ADC map right panel) as well as elder cortical and subcortical postischaemic lesions within the right hemisphere (**b**). TOF angiography revealed small arteria basilaris and bilateral occluded proximal arteria cerebri posterior (**c**), which were peripherally collateralized from anterior circulation. Since further diagnostic procedures were not fully conclusive, patient was planned for biopsy. Empiric treatment for vasculitis was initiated after non-diagnostic biopsy. Patient showed a clinical treatment response under empiric therapy and no new lesions in MRI at follow-up (**d**)

**Fig. 5:**
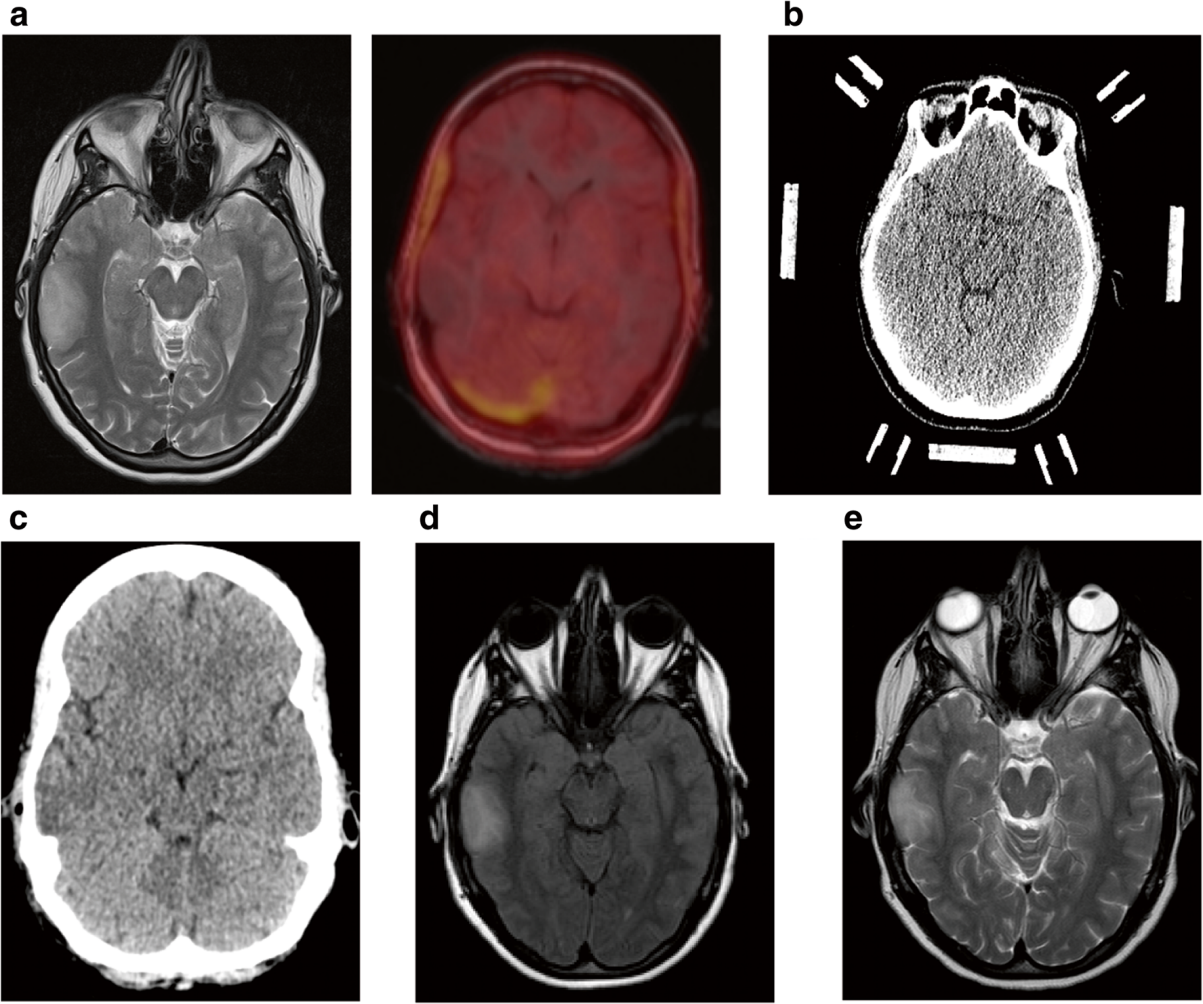
Patient was admitted for biopsy with a T2 diffuse hyperintense cortico-subcortical lesion within the right temporal lobe (**a**, left panel) showing no tracer enhancement in FET PET MRI (**a**, right panel) and no MRI contrast enhancement (not shown). Biopsy was planned with CT in a stereotactic frame (**b**). Postbiopsy CT showed no biopsy-associated complications (**c**). Since biopsy was non-diagnostic, follow-up was performed after 3 weeks (**d**) and 7 months (**e**) showing no change in MRI findings

Patients from the second group (*n* = 16, 37.2%) were empirically treated antiviral, antibacterial, or antimycotic agents or immunosuppression according to the suspected diagnosis provided by clinical and imaging findings. All patients with sufficient follow-up improved clinically (Fig. 4). A further group of 15 patients (34.8%), mainly patients without neurological deficits in a good clinical condition, underwent clinical follow-up and repeat cranial imaging (Fig. 5). We observed that the non-diagnostic biopsies did not affect the patient’s outcome adversely. These findings are similar to the study by Air et al. and Zoellner et al. [2, 32].

In cases of non-diagnostic biopsies, we recommend early postoperative imaging to confirm the intended biopsy location. Repeat biopsy should be performed when the target was missed. If the lesion was accessed and pathology is inconclusive, management depends on the suspected underlying diagnosis. In case of strong evidence for a tumor, re-biopsy or open resection depending on size and location of the mass should be considered. However, when primarily suspecting a neurological disorder, e.g., vasculitis or neurodegenerative disease, we recommend empiric treatment and re-evaluation, as the diagnostic yield for those lesions appears to be lower and the main contribution of the biopsy in those cases is the exclusion of a neoplasm [25]. In cases of clinical or morphological progression under therapy and/or lack of response to treatment, we recommend a repeat biopsy. Obviously, these rare cases are subject to interdisciplinary discussion on an individual case base taking the patients’ clinical and neurological status, other diagnostic findings, and the retrieved pathological results into account.

### Limitations

Due to the retrospective character of the study, it faces some limitations. Patient care was continued in several cases at the referring hospital, explaining a lack of long-term follow-up data of those cases. Patient selection and determination of various treatment options is an important factor when analyzing these results as the study overlooks a long period with several surgeons responsible for treatment algorithms. However, all cases were discussed in a multidisciplinary manner prior to surgery. There are likely more factors associated with non-diagnostic biopsies, e.g., the needle trajectory or angle of approach, that were not subject to analysis in this study. We recommend the inclusion of those factors in future prospective studies.

## Conclusions

We show that stereotactic biopsy is a safe procedure and provides a reasonable diagnostic yield even in cases with non-neoplastic lesions, when non-invasive diagnostic was inconclusive. Our study revealed that the likelihood of a non-diagnostic biopsy was significantly higher in patients with non-neoplastic lesions. Management of patients with inconclusive biopsies should be based on the initial assumption of the etiology of the lesion prior to surgery. In case of strongly suspected tumor, biopsy should be repeated and in case of suspected non-neoplastic lesion, such as inflammatory or neurodegenerative empirical treatment and/or close follow-up are recommended.
