# Correction to: Evaluation of 311 contemporary cases of stereotactic biopsies in patients with neoplastic and non-neoplastic lesions—diagnostic yield and management of non-diagnostic cases

**DOI:** 10.1007/s10143-021-01631-0

**Published:** 2021-08-28

**Authors:** Krystyna Agnieszka Pasternak, Michael Schwake, Nils Warneke, Max Masthoff, Samer Zawy Alsofy, Eric Suero Molina, Walter Stummer, Stephanie Schipmann

**Affiliations:** 1 Department of Neurosurgery, University Hospital Münster, Albert-Schweitzer-Campus 1, 48149 Münster, Germany; 2 Institute of Clinical Radiology, University Hospital Muenster, Münster, Germany; 3 Department of Medicine, Faculty of Health, Witten/Herdecke University, Witten, Germany; 4 Department of Neurosurgery, St. Barbara-Hospital, Academic Hospital of Westphalian Wilhelms-University Münster, Hamm, Germany

**Correction to: Neurosurgical Review**

**https://doi.org/10.1007/s10143-020-01394-0**

The article “Evaluation of 311 contemporary cases of stereotactic biopsies in patients with neoplastic and non-neoplastic lesions—diagnostic yield and management of non-diagnostic cases”, written by Krystyna Agnieszka Pasternak, Michael Schwake, Nils Warneke, Max Masthoff, Samer Zawy Alsofy, Eric Suero Molina, Walter Stummer, and Stephanie Schipmann, was originally published electronically on the publisher’s internet portal on September 20, 2020 without open access. With the author(s)’ decision to opt for Open Choice, the copyright of the article changed on July 6, 2021 to © The Author(s) 2020 and the article is forthwith distributed under a Creative Commons Attribution 4.0 International License, which permits use, sharing, adaptation, distribution and reproduction in any medium or format, as long as you give appropriate credit to the original author(s) and the source, provide a link to the Creative Commons licence, and indicate if changes were made. The images or other third party material in this article are included in the article's Creative Commons licence, unless indicated otherwise in a credit line to the material. If material is not included in the article's Creative Commons licence and your intended use is not permitted by statutory regulation or exceeds the permitted use, you will need to obtain permission directly from the copyright holder. To view a copy of this licence, visit http://creativecommons.org/licenses/by/4.0/. Open Access funding enabled and organized by Projekt DEAL.

The original article has been corrected.

